# Recent Achievements in Total Synthesis for Integral Structural Revisions of Marine Natural Products

**DOI:** 10.3390/md20030171

**Published:** 2022-02-25

**Authors:** Min Woo Ha, Jonghoon Kim, Seung-Mann Paek

**Affiliations:** 1 Jeju Research Institute of Pharmaceutical Sciences, College of Pharmacy, Jeju National University, 102 Jejudaehak-ro, Jeju 63243, Jeju-do, Korea; minuha@jejunu.ac.kr; 2 Interdisciplinary Graduate Program in Advanced Convergence Technology & Science, Jeju National University, 102 Jejudaehak-ro, Jeju 63243, Jeju-do, Korea; 3 Department of Chemistry, Soongsil University, 369 Sangdo-ro, Dongjak-gu, Seoul 06978, Korea; jhkim19@ssu.ac.kr; 4 College of Pharmacy and Research Institute of Pharmaceutical Sciences, Gyeongsang National University, 501 Jinju-daero, Jinju 52828, Gyeongnam-do, Korea

**Keywords:** marine natural product, total synthesis, structural revision, structure elucidation

## Abstract

A great effort to discover new therapeutic ingredients is often initiated through the discovery of the existence of novel marine natural products. Since substances produced by the marine environment might be structurally more complex and unique than terrestrial natural products, there have been cases of misassignments of their structures despite the availability of modern spectroscopic and computational chemistry techniques. When it comes to refutation to erroneously or tentatively proposed structures empirical preparations through organic chemical synthesis has the greatest contribution along with close and sophiscated inspection of spectroscopic data. Herein, we analyzed the total synthetic studies that have decisively achieved in revelation of errors, ambiguities, or incompleteness of the isolated structures of marine natural products covering the period from 2018 to 2021.

## 1. Introduction

Many researchers are involved in the search for new bioactive ingredients to treat a variety of diseases [1]. Drug candidates can be identified through the rational design of ligands that bind to disease-related proteins. Another strategy involves using natural products as inspiration for the chemical structure of drugs. To sustain life, living organisms biosynthesize substances with unique molecular structures and release these substances or their metabolites to defend against external stimuli. These compounds often demonstrate excellent antibacterial, antioxidant, antimicrobial, antiviral, and antitumor activities [2]. Furthermore, medicinal chemists have closely examined the chemical structures of natural products and derived molecules with improved functionality and stability through chemical synthesis [3].

These recently discovered molecules have been synthesized through developments in organic synthesis and ultimately used to develop new drugs [4]. In addition, advances in chromatography and spectroscopy have enabled the rapid and precise determination of the composition and chemical structures of numerous terrestrial and marine molecules [5]. Furthermore, with innovations in exploration technology that allow greater access to the ocean, a variety of new materials are rapidly being identified [6]. These previously unknown substances are then evaluated to determine their physiological activities and metabolic processes. The determination of the exact molecular structure of the substance is fundamental because the physicochemical and physiological properties of the substances are often influenced by the internal spatial arrangement of atoms [7]. However, sometimes structural determination using chromatographic and spectroscopic analysis is inadequate. Errors can occur, and sometimes, unlikely structures are proposed. Most errors are identified through direct comparison with the physicochemical data of synthesized versions of the compounds. The accurate chemical structures of the natural products were then elucidated through preparation of the substances via synthetic chemistry [8].

Discrepancies often arise due to constitutional misassignments or inaccurate stereochemistry judgments [9]. The former are related to chronic confusion between 5- and 6-membered rings, overlooking of double bond configuration and regioisomerism, and misjudgment of the symmetry related to dimerization. The latter implies rival configurational errors. According to reviews, structural misassignments and subsequent revisions of the structure of these natural products occur with a remarkably high frequency. In 2005, Nicolaou and Snyder were concerned that the errors in structural determination could result in chain errors when proposing the biosynthetic pathway or metabolic process of natural products and stated that the results could lead to unnecessary expenses [10]. Later, Maier discussed structural modifications of natural products reported between 2005 and 2009 [11]. In 2009, Usami presented examples of structural revisions for several marine natural products, including the discrepancies they identified during the total synthesis of pericosines [12]. In each case, the organic synthesis was presented in a way that demonstrated the usefulness of the total synthetic study. In 2011, McPhail performed a statistical analysis of the techniques used in structural misassignments and subsequent revisions from 2005 to 2010 [13]. Updated records on structural reassignments of natural products are available through a recent review by Kubanek from 2018 [14]. In addition, we encountered many cases that require critical re-assessment for structural identification of natural products of marine origin. Table 1 shows the marine natural products whose structural errors were verified between 2018 and 2021. The authors of the papers that directly contributed to this work are listed in the columns labeled isolation and revision. Two types of molecular masses—one was the initially proposed structure, and the second was the genuine compound with the corrected structure—were actually on the hands of the researchers, with the exception of entries 6, 9, and 17 in Table 1. For entries 6 and 9, X-ray crystallography have justified reassignments of haliclonadiamine and certain julichromes. Extensive dissection of 1D and 2D NMR datasets combined with X-ray crystallography enabled rigorous elucidation of the previously reported configurations of marine natural products. For wentiquinone C in the entry 17, it’s the case that the actual skeleton of this material has been asserted to be a xanthone, not an anthraquinone. Wang group has ever methylated this natural substance in order to find more bioactive secondary metabolites from the same fungal strain, and figured out this methylated one was calyxanthone (already known in 1983). In this study, we summarize and explain the contributions of synthetic chemistry to identifying and rectifying errors in natural product structure elucidation.

## 2. Examples of Total Synthesis of Marine Natural Products Resulting in Their Structural Revisions

### 2.1. Synthesis of Boholamide A

**Scheme 1:**
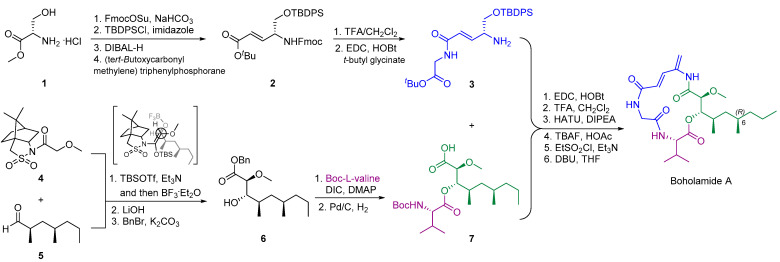
Synthetic route for preparation of the actual structure of boholamide A.

In 2020, the Schmidt group isolated a new substance from a *Truncatella* sp. mollusk and elucidated its chemical structure [35]. They named the substance boholamide A after the island in the Philippines where the original material was collected. In terms of its cytotoxicity, the IC_50_ value of boholamide A was determined to be within the concentration range of 100–400 nM. It was therefore expected that this new substance could contribute to the development of novel anticancer compounds for use as potential chemotherapeutic agents. However, due to its limited availability from marine resources, further investigations into the physiological activity of boholamide A were limited. Thus, based on its potential biological activity and limited natural availability, it became a target of synthetic chemists. As a result, in 2021, the Chen and Zhang group reported the first total synthesis of boholamide A based on a previously proposed chemical structure [15]; however, some of the spectral data of the synthesized molecules (i.e., the chemical shift of the C6-H at ^1^H-NMR spectrum and the ^13^C-NMR peaks corresponding to the C-2, 3, 4, 6, 7, and 9 atoms) were found to differ from those of the natural product. Under the hypothesis that the chirality of the C-6 position was the cause of this discrepancy, the same group synthesized an epimer in which the stereochemistry of the C-6 center was inverted. The resulting ^1^H-NMR spectrum was identical to that of the natural product; however, the ^13^C-NMR chemical shifts of the two methyl groups of the valine residue differed slightly from the reported values. Despite these differences, it was not unreasonable to consider that this C-6 epimer was the most plausible structure to date. Thus, after approximately 1 year and 3 months, a revision of the structure of this new anticancer molecule was completed following its preparation as described below (Scheme 1).

Boholamide A contains a 15-membered macrocycle and is a depsipeptide, i.e., a peptide in which one or more of the amide groups is replaced by the corresponding ester. This substance is characterized by possessing the 4-amino-2,4-pentadienolate (APD) moiety; however, unlike some other natural products containing this moiety [36,37,38,39,40,41,42,43,44], boholamide A bears a methoxy group at the 2-position. As shown in Scheme 1, the synthetic process produced two molecular fragments, which are colored in blue, **3** and green, **7**. More specifically, L-serine methyl ester (**1**), which can be easily obtained, was used as a starting material to obtain the blue fragment; Fmoc *N*-hydroxysuccinimide ester was used to protect the amine group, while the TBDPS-Cl was used to protect the alcohol group through the formation of a silyl ether. After reducing the methyl ester to an aldehyde moiety using DIBAL, a classical Wittig reaction was carried out to produce the *E*-isomer of the olefin. The previously incorporated *t*-butyl ester, **2** was then hydrolyzed under acidic conditions to give the carboxylic acid, which was coupled with glycine *t*-butyl ester to generate an amide bond, while the free amine was produced by removal of the Fmoc group. To prepare the green fragment, **7** a chiral auxiliary was employed along with a Mukaiyama aldol reaction [45]. The active methylene substrate, **4** bearing the camphorsultam unit was converted to its corresponding silyl enol ether by TBSOTf [46], which was added to the desired aldehyde (possessing the desired chirality) in the presence of boron trifluoride etherate. This process allowed the stereoselective introduction of a methoxy group at the C-2 position, in addition to C-O bond formation at the C-3 position of boholamide A. After removal of the chiral auxiliary, a benzyl ester, **6** was obtained, and the free hydroxyl group of the molecule was combined with Boc-L-valine to construct an ester; the benzyl protecting group was removed while simultaneously converting it to toluene. The obtained fragment, **7** was then combined with the previously prepared blue fragment, **3** to form an amide. More specifically, following deprotection for the terminal carboxylic acid and amine groups, a subsequent HATU-promoted intramolecular cyclization coupled the two fragments together to construct a 15-membered ring. Finally, the total synthesis of boholamide A was completed by generating an external olefin through subsequent reactions.

### 2.2. Synthesis of Chrysamide B

Chrysamide B is a molecule containing a chiral piperazine scaffold, and was first reported following its isolation from the deep-sea-derived fungus *Penicillium chrysogenum* SCSIO41001 in 2016 [47]. Liu et al., who isolated this substance and proposed its chemical structure for the first time, were surprised to find that it was structurally biosynthesized from D-alanine, despite the fact that natural amino acids are generally found in their L-form [48,49]. A year later, the Voyer group attempted the total synthesis of this new molecule due to the pharmacological interest surrounding the piperazine scaffold [50]. They reported that their initially proposed chemical structure of chrysamide B could be obtained by synthesizing a chiral epoxyacid through a Sharpless-Katsuki epoxidation following the construction of a piperazine core by solid-phase peptide synthesis using a D-alanine derivative as a substrate. However, at this point, they were unaware of the issues related to the stereochemistry of this unprecedented chemical structure. In 2018, the Wang group explored the syntheses of chrysamides A and B and investigated their antibacterial activities. During their studies, it was cautiously suggested that chrysamide B may be generated from the L-form of the amino acid, as is usually the case with natural products. Accordingly, following its successful preparation, they confirmed that the spectral data were consistent with those of the natural compound, and this claim was supported by X-ray crystallographic analysis [16].

**Scheme 2:**
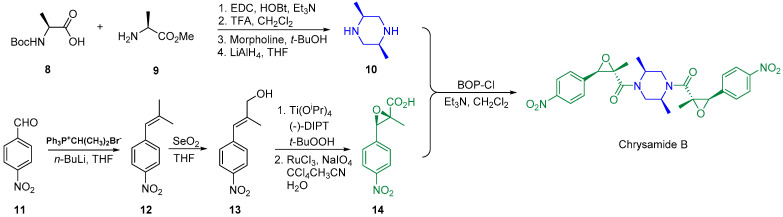
Synthetic route for preparation of the actual structure of chrysamide B.

The synthetic process of this unequivocally determined chrysamide B structure began with the condensation of two alanine derivatives, **8** and **9**, in the L-form. The resulting dipeptide was converted to its corresponding diketopiperazine via a recombination with the deprotected residues. Subsequently, a piperazine skeleton of **10** possessing the intended configuration was prepared by reaction with an excess of LiAlH_4_. A Wittig reaction and three successive oxidation reactions were then employed to obtain the epoxy-acid fragment, **14**. Following a Riley oxidation of **12** using selenium dioxide [51], the allylic alcohol, **13** was converted to a chiral epoxy-alcohol via a Sharpless asymmetric epoxidation [52], and the green fragment, **14** (Scheme 2) was obtained in good conversion by oxidation in the presence of NaIO_4_ and RuCl_3_ [53]. The authors found that the assembly of these fragments was best mediated by BOP-Cl. The obtained chrysamide B, whose structure was confirmed to be identical to that of the natural product, is now being used to explore its role in Nature.

### 2.3. Synthesis of Dysiherbol A

Dysiherbol A is an avarane meroterpenoid that exhibits anti-inflammatory and anticancer effects. In 2016, it was newly isolated from a sponge called *Dysidea* sp., which was found in the South China Sea [54]. This compound contains a number of interesting structural features, and thus was a challenge for synthetic chemists. In 2021, two independent research groups successfully prepared this compound, which led to the assertion that the chemical structure initially proposed by Jiao et al. was not indeed the correct structure. More specifically, dysiherbol A was initially thought to be a tetracyclic carbon ring system containing A, B, C, and D rings. However, it was later found that the hydroxyl group at the C-4 position of the A ring and the hydroxyl group at the C-20 of the D ring (see Table 1, entry 3) were fused to form a pentacyclic skeleton containing an ether bond. Subsequently, the Schmalz [17] and Liu [18,19] groups individually synthesized the confirmed structure of dysiherbol A for the first time, although different synthetic routes were employed.

**Scheme 3:**
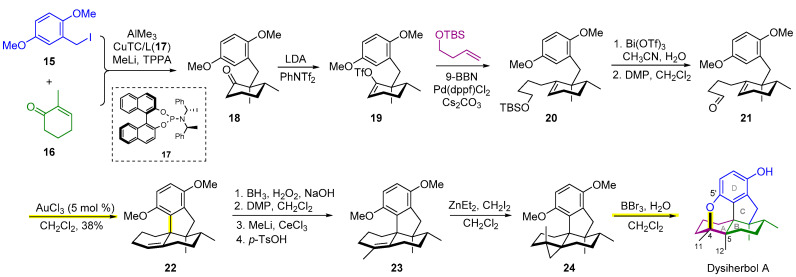
Synthetic route for preparation of the actual structure of dysiherbol A by Schmalz.

Scheme 3 shows the asymmetric route employed by the Schmalz group, and this synthesis is characterized by the use of a unique gold-catalyzed double cyclization reaction [55,56,57,58,59]. More specifically, their preparation commenced with the stereoselective methylation of 2-methyl-2-cyclohexenone, **16** via a Cu-mediated Michael addition in the presence of a Lewis acid and chiral ligand, **17** [60]. In this reaction system, benzyl iodide, **15** which possesses two methoxy groups, was used to react in situ with the activated enolate. The obtained single diastereomer of the ketone, **18** was then converted into its corresponding enol triflate, **19** which was connected via a Suzuki-Miyaura coupling reaction with the alkene group of the fragment that will form the final A ring [61]. Following deprotection of the silyl ether of **20**, the resulting alcohol was oxidized for later use. While searching for reaction conditions that simultaneously generated the A and C rings, the authors found that only AuCl_3_ (in a manner different from general Lewis acids) exhibited a unique and extraordinary performance [62]. In particular, no significant sensitivity to air or moisture was observed. Methylation of the C-4 and C-5 positions of the obtained tetracyclic olefin was then achieved through a challenging trial and error approach. More specifically, the optimal approach involved introduction of the C-4 methyl group via a 1,2-addition of a ketone moiety and continuous H_2_O elimination. Subsequently, the C-5 methyl group (which becomes the C-12 position) was introduced via a Simmons-Smith cyclopropanation reaction and acidic cleavage [63]. This route represented a meaningful research achievement in which the chemical structure of dysiherbol A was confirmed, and the absolute configuration was clearly elucidated.

**Scheme 4:**
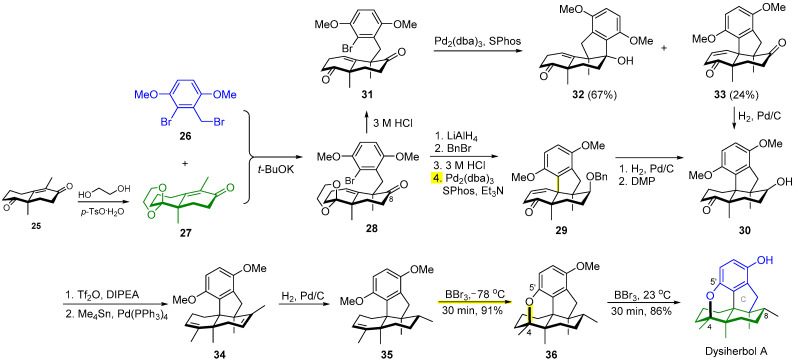
Synthetic route for preparation of the actual structure of dysiherbol A by Lu.

The synthesis of dysiherbol A by the Liu group [18,19] was characterized by a route that constructs a 5-membered ring, namely the C ring (Scheme 4). During this process, the authors experienced some issues related to the methyl group at the C-8 position. As shown in Scheme 4, the Wieland-Miescher ketone derivative, **27** reacted well with the corresponding aryl bromide, **26** to give intermediate **28**, which appeared suitable for cyclization to produce the 5-membered ring [64]. However, the authors initially attempted the intramolecular cyclization of a compound in which the ketone group of intermediate **28** was replaced with a methyl group, but they found that this reaction did not proceed even after several attempts. It was speculated that this reaction failed due to the presence of a methyl group, and so its introduction was delayed. Thus, using a benzyl ether, the intended Heck reaction proceeded smoothly, and a 5-membered ring was generated. Following subsequent removal of the benzyl ether under reducing conditions, the intermediate was converted to its corresponding enol triflate and reacted with Me_4_Sn to introduce a methyl group at the C-8 position [65]. Finally, the methoxy group of the aryl ring of **36** was converted to the free alcohol in the presence of BBr_3_ to obtain the desired dysiherbol A.

### 2.4. Synthesis of Echinosulfone A and Reconsideration of Structures of Echinosulfonic Acid A~D

**Scheme 5:**
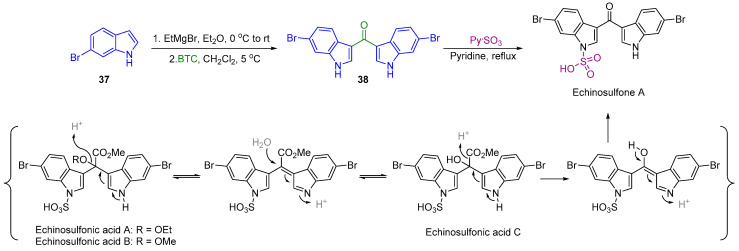
Synthesis of actual echinosulfone A and reconsideration of the structures of echinosulfonic acids A–D.

Echinosulfone A was first isolated from a southern Australian marine sponge by the Capon group in 1999 [66], although its structure was not correctly elucidated until 2020. Following this declaration in 2020 regarding their initially proposed but flawed structure, the Carroll group presented objective evidence based on their synthetic studies, which explained that there was an error in the arrangement of the functional groups in the chemical structure of echinosulfone A [20]. More specifically, this dibrominated bis-indole alkaloid was prepared without difficulty over three steps (Scheme 5). Initially, two equivalents of an indole Grignard reagent [67] were linked by bis(trichloromethyl)carbonate (BTC, triphosgene) to produce bis(6-bromo-1*H*-indol-3-yl)methanone (**38**) [68]. Following sulfonation on only the nitrogen atom using a pyridine sulfur trioxide complex, the authors were able to obtain new fragmentation data from mass spectrometric analysis. Their findings led to a reasonable suspicion that the chemical structures of echinosulfonic acids A–D were not also correctly elucidated. Using the biosynthesis process of echinosulfonic acids A–D as inspiration, the authors re-examined the spectroscopic data of these natural products and proposed a modification of their chemical structures [21].

### 2.5. Synthesis of Halioxepine

Halioxepine is a new meroditerpene substance that was isolated from an Indonesian *Haliclona* sp. sponge in 2011 [69]. At the time of the initial publication, it was noted that the chemical structure remained unclear, although a proposed structure was given. More specifically, the spin interaction between the two stereoclusters, i.e., the hydroquinone-tetrahydrooxepine region and the cyclohexene region, had not been clearly identified. Therefore, Tanaka and co-workers [69] suggested that the absolute stereochemistries of the C-10 and C-15 centers could be (10*S*, 15*S*) or (10*R*, 15*R*), although no definitive determination was carried out. Seven years later, in 2018, the Rodriguez group reported halioxepine B and C, which are congeners of halioxepine, and claimed that the relative configuration was thought to be (1*S*, 2*S*, 7*R*, 10*R*, 15*R*) based on NMR and computational chemistry results [70]. However, to clear up any confusion related to the structure and configuration, Poock and Kalesse directly compared the spectral results obtained for the natural and synthetic compound through the total synthesis of halioxepine, and it was found that the Kalesse group had synthesized (−)-*ent*-halioxepine, an enantiomer of the natural product halixepine [23].

**Scheme 6:**
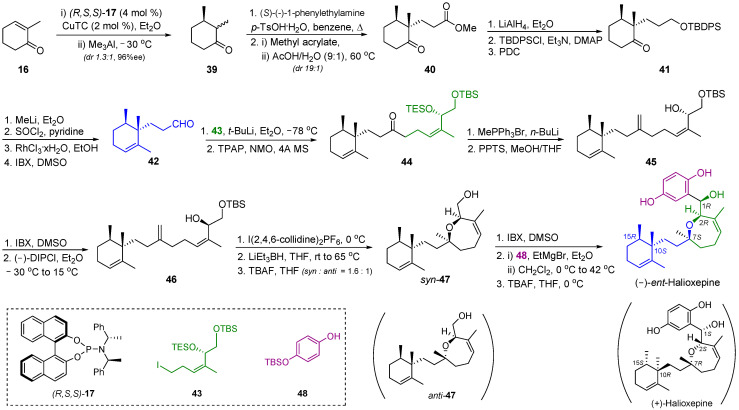
Synthetic route for preparation of the actual structure of halioxepine.

Thus, the synthesis carried out by the Kalesse group began with the preparation of 2,3-dimethylcyclohexanone, **39** through a Cu-mediated asymmetric 1,4-addition reaction (Scheme 6). The stereoselectivity induced by this reaction was poor [71]; however, this was inconsequential since subsequent enamine formation and the Michael addition of methyl acrylate generated the (10*S*, 15*R*) chirality [72]. Following reduction of the ketone, **40** by LiAlH_4_ and reaction with pyridinium dichromate, methyl addition was carried out and a mixture of endo and exo olefins was obtained via a subsequent elimination step. This mixture was then reacted with rhodium(III)-chloride to promote isomerization into an internal alkene within the ring and conversion of the silyl ether into an alcohol [73]. Oxidation using 2-iodoxybenzoic acid produced an aldehyde, **42** which was subsequently employed in the production of the tetrahydrooxepine segment of the halioxepine structure. Iodide compound **43**, which contained 7 carbon atoms, acted as a nucleophilic synthon following halide–metal exchange, and was thus added to the aldehyde, **42**. Olefination with MePPh_3_Br then led to the generation of external olefin **45**. Subsequently, the researchers skillfully inverted the stereochemistry of the hydroxyl group in compound **45** through oxidation followed by a stereoselective reduction [74], and formation of the 7-membered ring tetrahydrooxepine was completed in a good yield upon treatment with bis(2,4,6-trimethylpyridine)iodine(I) hexafluorophosphate. Finally, the desired halioxepine bearing the correct relative configuration was prepared via a number of subsequent uneventful reactions.

### 2.6. Synthesis of Iriomoteolide-2a

Iriomoteolide-2a was first reported by the Tsuda group in 2015 [75]. It is a new ingredient found in the culture medium of marine dinoflagellate *Amphidinium* sp. (HYA024 strain) collected from Iriomote island in Okinawa, Japan, and possesses a remarkable cytotoxicity and antitumor activity. This substance is composed of a 23-membered macrolactone backbone containing a bis(tetrahydrofuran) ring. In 2018, Fuwa reported the first synthesis of a compound possessing the chemical structure proposed by Tsuda; however, it was found that the ^1^H- and ^13^C-NMR spectra of the synthesized compound were different from those of the natural product [24]. Recognizing that the structure must be modified, the authors investigated the ROESY and HSQC-NOESY correlations for this structure, and carried out a *J*-based configuration analysis [76]. After revising the initial claim that the relative configuration of the C-12/C-13 segment in iriomoteolide-2a is the *threo* configuration, they hypothesized that it could indeed be the *erythro* configuration, and this was supported by the following synthetic study.

**Scheme 7:**
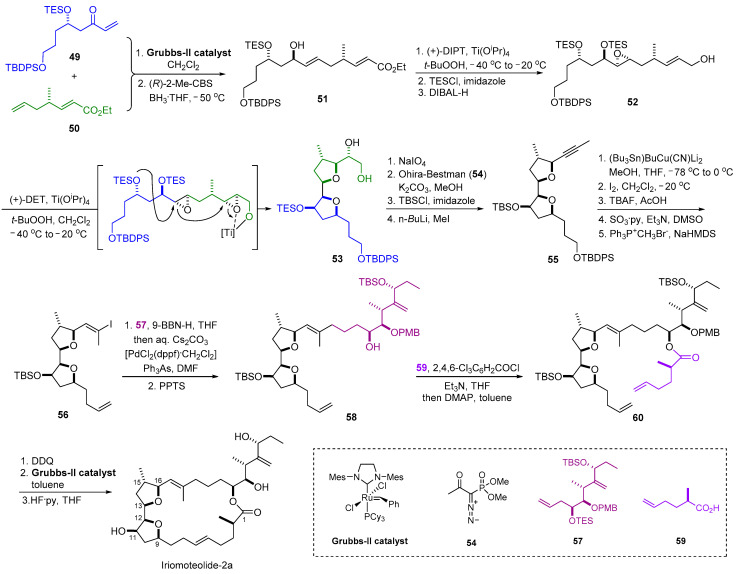
Synthetic route for preparation of the actual structure of iriomoteolide-2a.

The key step in the synthesis of iriomoteolide-2a is the stereo-controlled synthesis of the bis(tetrahydrofuran) unit, and this was achieved through an asymmetric epoxidation/diepoxide cyclization cascade. The overall synthetic process began with an olefin cross-metathesis promoted by a Grubbs II catalyst [77], as outlined in Scheme 7. The use of borane with a chiral oxazaborolidine as the catalyst led to the production of a chiral hydroxy group of **51** [78], and subsequently, the diepoxide produced by a double Sharpless asymmetric epoxidation process was cyclized to furnish the desired bis(tetrahydrofuran) backbone [79]. The 1,2-diol, **53** was then converted to an aldehyde via oxidative cleavage using sodium periodate, and the aldehyde was converted into an alkyne using the Ohira-Bestman reagent, dimethyldiazomethylphosphonate (**54**) [80]. The triethyl silane (TES) group that underwent desilylation during treatment with sodium periodate was replaced by the TBS group following generation of the alkyne. The terminal alkyne was then methylated to afford **55**. Stannylcupration of **55** followed by iodolysis, chemoselective deprotection of the silylether, oxidation of the free alcohol, and subsequent methylenation afforded the vinyl iodide backbone [81], which could be applied in a Suzuki-Miyaura coupling reaction. More specifically, following the chemoselective hydroboration of compound **57** using 9-BBN, coupling with the prepared vinyl iodide, **56** was carried out. The TES group was subsequently removed, and the obtained free secondary alcohol was esterified with **59** under Yamaguchi reaction conditions [82]. Following removal of the *p*-methoxybenzyl group using DDQ and ring-closing metathesis in the presence of a Grubbs II catalyst [73], a 23-membered ring was obtained. Finally, deprotection under acidic conditions afforded the desired iriomoteolide-2a for the first time.

### 2.7. Synthesis of Keramamide A & L and Mozamamide A

Keramamides A and L were isolated from the marine *Theonella* sponge in 1991 [83] and 1996 [84], respectively, as reported by the Kobayashi group. Both are anabaenopeptin-type peptides containing the pentapeptide group as their main structural feature. Within this structure, an amide bond is formed by the ε-nitrogen atom of the N-terminal lysine residue, while the α-nitrogen atom of the lysine amino acid is linked with another amino acid in the form of a urea. Although both keraminde A and L have this characteristic in common, only the former contains a hydroxyl group in its indole ring. Interestingly, this results in quite different cytotoxicity profiles.

**Scheme 8:**
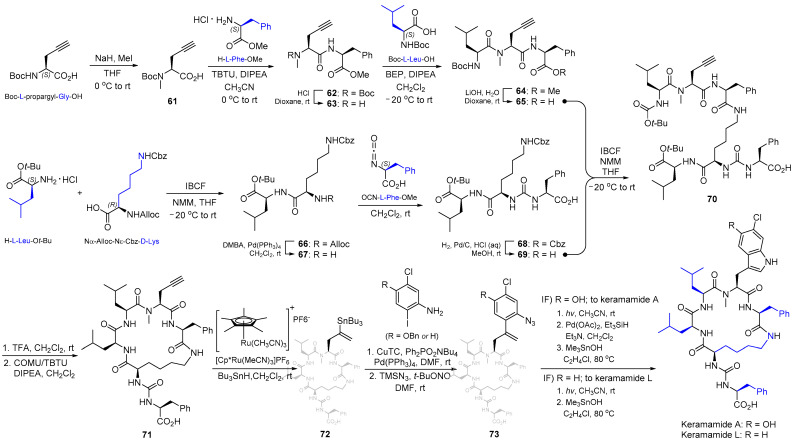
Synthetic route for preparation of the actual structures of keramamides A and L.

To obtain these substances, many synthetic chemists have adopted the strategy of attaching the indole ring as a substituent in the final synthetic step (Scheme 8). According to this strategy, the Kazmaier group, who were the first to report the synthesis of these molecules, initially prepared and connected two different tripeptides [26]. The first tripeptide, **65** was synthesized by linking an α-propargylated *N*-methylglycine derivative, **61** in the *S* configuration with L-phenylalanine and L-leucine. The second tripeptide, **69** was synthesized by linking the dipeptide obtained by combining L-leucine and D-lysine with L-phenylalanine in the form of isocyanate. The first tripeptide, **65** was then linked to the free ε-nitrogen atom of the lysine residue of the second one, **69**. After the generation of a large ring upon amide bond formation, the effort to install the substituted indole core began. Thus, the propargyl group was converted to the required vinyl stannane via a hydrostannation approach, the corresponding aryl ring was introduced by a Stille cross-coupling reaction, and the azide group on the aryl ring introduced by an azidation-induced photo-chemical nitrene C-H insertion into a nearby olefin [85,86]. As a result of this successful first total synthesis, it was found that D-form lysine, not L-form lysine, was involved in the synthesis of keramides A and L. During the numerous amide bond formation reactions that were performed during the preparation process, the following coupling reagents and activating reagents were employed: TBTU [2-(1*H*-benzotriazole-1-yl)-1,1,3,3-tetramethylaminium tetrafluoroborate], BEP (2-bromo-1-ethyl-pyridinium tetrafluoroborate), IBCF (isobutyl chloroformate), and COMU [(1-cyano-2-ethoxy-2-oxoethylidenaminooxy)dimethylamino-morpholino-carbenium hexafluorophosphate].

Mozamide A was isolated and reported by the Faulkner group in 1997 [87]. More specifically, it was isolated from a sponge of the genus *Theonella* collected in Mozambique, and structurally, it is quite similar to keramides A and L, with the exception that it does not possess a chlorine atom in the tryptophan side chain, and it possesses a D-valine residue instead of L-leucine. In the case of mozamide A, the amino acid bound as the urea form was L-allo-isoleucine. The Kazmaier group, who were the first to synthesize and characterize keramamides A and L corrected the structural error in mozamide A by the first total synthesis in the same manner a year later [27].

### 2.8. Synthesis of Mytilipin B

The chlorosulfolipids (CSLs) are a family of natural marine lipids [88]. Unlike typical biological lipids, they contain hydrophilic sulfate and chloride groups in the hydrocarbon tail. Mytilipin B was first isolated in 2002 from the culinary mussel *Mytilus galloprovincialis* [89], and the stereochemistry of mytilipin B, which was initially unclear, was correctly assigned by the Carreira group in 2019. Mytilipin B contains a total of 15 chiral centers, and the previous misassignments of eight stereocenters were corrected by this group through the step-by-step preparation of four diastereomers.

**Scheme 9:**
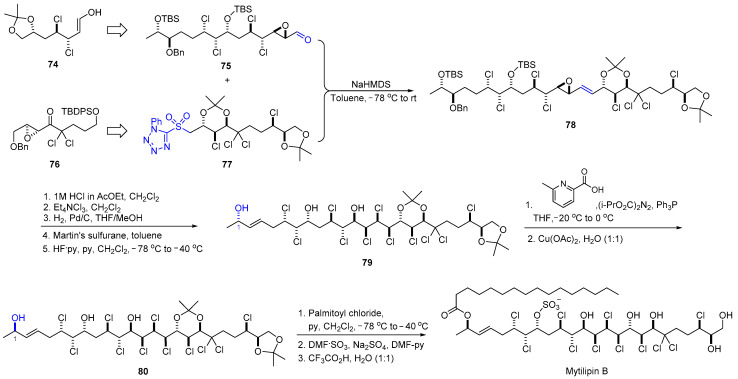
Synthetic route for preparation of the actual structure of mytilipin B.

More specifically, their journey to determining the correct structure among the 32,768 possible stereoisomers was extremely detailed and systematic. They prioritized and implemented the synthesis based on the limited spectral data available from the natural products. The key to their synthetic route was to place the chlorine atoms and hydroxy groups in their intended arrangements along the backbone of the hydrocarbon chain. This was mainly achieved using asymmetric epoxidation and olefination reactions. More specifically, as shown in Scheme 9, the Julia-Kociensky olefination reaction was initially used to combine aldehyde **75** with sulfone **77** [90]. Aldehyde **75** had been previously prepared from **74** via 10 steps, including Sharpless asymmetric epoxidation, esterification, hydrolysis, silyl ether protection, selective desilylation, Dess–Martin periodinane (DMP) oxidation, and Wittig olefination reactions, followed by a final dichlorination using Mioskowki’s reagent (Et_4_NCl_3_) [91]. Sulfone **77** was obtained from **76** over 13 steps, including epoxide opening, reduction, acetal protection, desilylation, DMP oxidation, Stille-Gennari olefination, DIBAL reduction, and Sharpless asymmetric epoxidation reactions, which were followed by selective chlorolysis of the *cis*-2,3-epoxy alcohols, a Mitsunobu reaction, and final oxidation to the sulfone [92]. The preparation of **78** was then achieved by inserting chlorine atoms and hydroxyl groups at the desired positions of the olefin regions of intermediate **78,** where the two skeletons are connected. A subsequent Mitsunobu inversion allowed the chirality at C-1 to be inverted. In addition, it was found that the conversion rate was the highest when 6-methyl-2-picolinic acid was used during the search for reaction conditions. In the subsequent ester hydrolysis, it was fortunate that no chlorohydrin reactions were observed when (CHCl_3_/MeOH 1:1) and Cu(OAc)_2_ (10 equiv) were used. After successfully installing the intended stereocenters throughout compound **78**, mytilipin B was obtained through palmitoylation, *O*-sulfation, and acetonide deprotection.

### 2.9. Synthesis of Nocarbenzoxazole G

In 2019, the Ham group developed an efficient and practical method for the construction of the 2-arybenzoxazole framework [29]. Their syntheses of various related subtypes in this way using different aryl rings revealed that the previously reported chemical structure of the natural product nocarbenzoxazole G was incorrect. Nocarbenzoxazole G was isolated from the marine-derived halophilic bacterial strain *Nocardiopsis lucentensis* DSM 44048 in 2015 [93]. Structurally, it contains a methoxy phenol ring attached to the 2-position of the benzoxazole skeleton core. However, a previous report of the structure appeared to contain an error regarding the position of the methoxy group attached to the phenol ring.

**Scheme 10:**
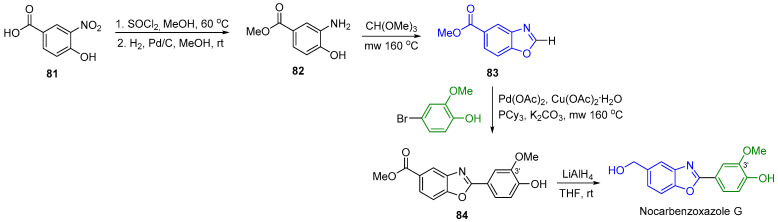
Synthetic route for preparation of the actual structure of nocarbenzoxazole G.

Thus, the Ham group developed the synthetic route shown in Scheme 10, which involved the conversion of 4-hydroxy-3-nitrobenzoic acid to its corresponding methyl ester and the generation of an aniline structure via the reduction of a NO_2_ group [94,95]. After dissolving the prepared benzoate **82** in trimethyl orthoformate and applying microwave irradiation (reaction temperature = 160 °C), 2*H*-benzoxazole, **83** was obtained in 30 min without the use of other reagents. After treating this material with 4-4-bromo-2-methoxyphenol, Pd(OAc)_2_, Cu(OAc)_2_·H_2_O, triphenylphosphine (PPh_3_), and K_2_CO_3_ in toluene under microwave irradiation at 160 °C for 30 min, the CH groups of the heteroatom ring was activated, and aryl-aryl coupling took place. Following a subsequent reduction of the ester of **84** to give an alcohol, nocarbenzoxazole G was obtained (Scheme 10).

### 2.10. Synthesis of Solomonamide A and B

Solomonamides A and B are macrocycles that were isolated from the marine sponge *Theonella swinhoei* in 2011 by the Zampella group [96]. Solomonamide A was found to exhibit a significant anti-inflammatory activity; however, the amount of solomonamide B obtained from the natural resource was too small to allow sufficient activity screening. Comparing their chemical structures, solomonamide A appears to be biogenetically derived from solomonamide B because the arrangement of all atoms is identical except for the presence or absence of a hydroxy group at the C-5 position. The Reddy group completed the syntheses of solomonamides A and B in 2018 [30] and 2016 [31], respectively, and correctly determined both chemical structures.

**Scheme 11:**
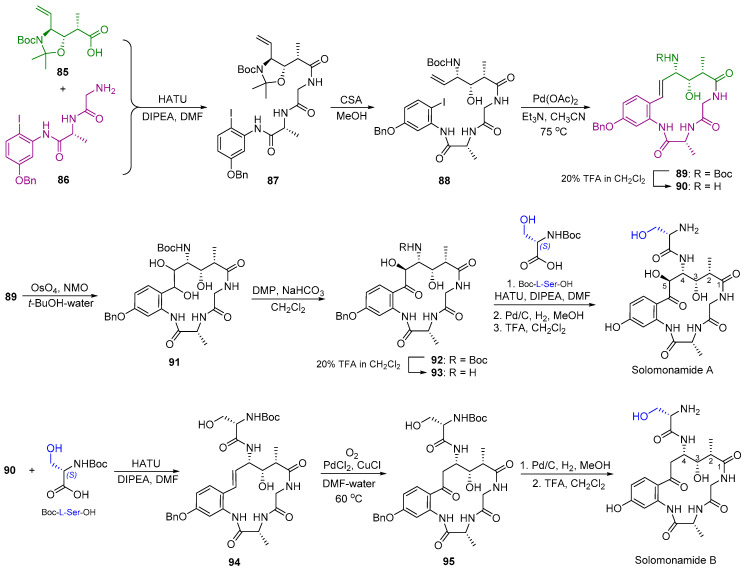
Synthetic route for preparation of the actual structures of solomonamides A and B.

Initially, they performed a total synthesis based on the first proposed chemical structures of the natural products and recognized that the spectral data of the synthesized molecules were different from those of the natural products. Thus, the systematic preparation of a stereoisomer with a new chemical structure was then carried out. As shown in Scheme 11, chain elongation was carried out using starting material **85** (prepared from L-methionine aldehyde [97])**,** in which the arrangement of the chains at the C-2, 3, and 4 positions of solomonamide A/B was already determined. A diastereoselective *syn*-aldol adduct was obtained using an Evans auxiliary, and methyl sulfane was converted to the terminal olefin through ozonolysis followed by reaction with CaCO_3_ in 1,2-dichlorobenzene. The first key reaction of Reddy’s total synthesis involved a ligand-free intramolecular Heck reaction [98], in which cyclic compound **89** was produced in a 48% yield using a highly diluted reaction solvent system (0.0015 M) in the presence of Pd(OAc)_2_ and Et_3_N. The second characteristic reaction was a regioselective oxidation reaction. In the case of solomonamide B, they succeeded in introducing an oxygen functionality only at the benzylic carbon atom through a Wacker-type oxidation of **94** [99] after linking serine to the core intermediate **90** without difficulty. Following a subsequent deprotection of **95**, the intended arrangement of solomonamide B was obtained, which was confirmed to be consistent with the spectroscopic results obtained for the natural product. The resynthesis of solomonamide A was then carried out maintaining the C-2, 3, and 4 positions constant and focusing on the introduction of a hydroxyl group at the C-5 position. Importantly, the authors established a strategy to oxidize only one hydroxyl moiety following the dihydroxylation of **89**. However, this was a slow process, since the general Sharpless dihydroxylation reaction of **89** was not successful. Surprisingly a single diastereomer of the diol was obtained under the Upjohn hydroxylation reaction conditions, and the chirality at the C-5 position was introduced through benzylic oxidation using Dess–Martin periodinane, and solomonamide A prepared via the appropriate modification of this intermediate. Following the preparation of both solomonamides A and B, it was demonstrated that their physicochemical and physiological activity values were consistent with those of the natural products (Scheme 11).

### 2.11. Synthesis of Peribysin A, B, C, F, and G

Among the substances belonging to the peribysin family, peribysins A–J were obtained from *Periconia byssoides* OUPS-N133 of the sea hare *Aplysia kurodai*. Numata and Yamada contributed significantly to the discovery and verification of these molecules [100,101]. As the expectation that the peribysins could act as cell adhesion inhibitors became known, these substances and their derivatives began to receive growing research attention. In terms of the construction of this interesting bicyclic system, the Danishefsky group reported the complete synthesis of peribysin E in 2007 [102]; however, their proposed absolute configuration has since been officially revised based on subsequent evidence. In addition, the total syntheses and structural revision of peribysins A, B, C, F, and G were later carried out by the Kulkarni and Reddy groups in 2020 [32].

**Scheme 12:**
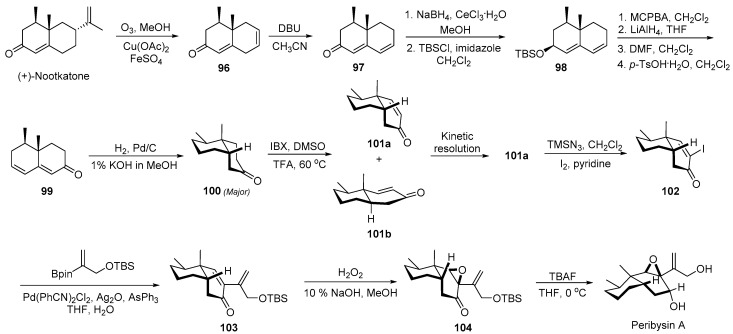
Synthetic route for preparation of the actual structure of peribysin A.

Following the successful preparation of peribysin A, several functional group manipulations yielded peribysins B, C, F, and G. Scheme 12 shows the method employed to obtain peribysin A starting from (+)-nootkatone, wherein the various stages were generally carried out using the crude mixtures (i.e., without the purification of intermediates). More specifically, the ozonolysis of (+)-nootkatone in methanol followed by treatment with soluble Cu(II) and Fe(II) tetrafluoroborate salts gave the desired absolute stereochemistry, and an alkene was generated following the subsequent removal of isoprene. This methodology was developed by Schreiber, Čeković, Newhouse, and others [103,104,105]. During this process, the peroxide O–O bond of the intermediate methoxy hydroperoxide species generated upon ozone injection is cleaved by Fe(II)/Cu(II) and then converted to an alkene by β-elimination. After double bond migration of **96** in the presence of 1,8-diazabicyclo(5.4.0)undec-7-ene (DBU), a Luche reduction using cerium(III) chloride hydrate was performed followed by alcohol protection to obtain compound **97**, wherein the newly introduced chirality was reported tentatively [106]. The epoxide obtained by the epoxidation of an unhindered alkene using *meta*-chloroperoxybenzoic acid was then regioselectively opened under reducing conditions to introduce the alcohol functionality, which was oxidized to a ketone using Dess–Martin periodinane. The silyl-deprotected hydroxyl group was eliminated using *para*-toluenesulfonic acid, during which double bond migration occurred to give **99**. Various conditions were examined to obtain a junction in which the two cyclohexane rings were connected in a *cis* geometry, with hydrogenation in a 1% methanolic solution of KOH being found to be the most effective. Although two enones (**101a** and **101b**) were obtained through the 2-iodoxybenzoic acid oxidation protocol, a single isomer, **101a** was recovered following kinetic resolution by iodination with iodine in pyridine. As a result, the halide was attached to the almost completed core skeleton, thereby allowing Suzuki coupling with the appropriate boronate ester to be carried out [107]. Subsequent epoxidation and deprotection of the internal ring alkene finally yielded peribysin A with the correct chemical structure, as confirmed by single-crystal X-ray analysis.

**Scheme 13:**
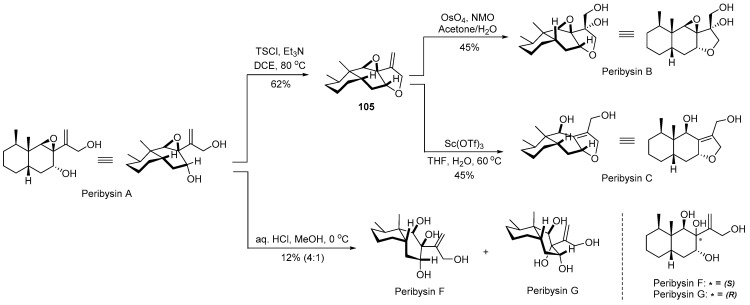
Synthetic route for preparation of the actual structures of peribysins B, C, F, and G.

Scheme 13 outlines the preparation of peribysins B, C, F, and G from peribysin A. More specifically, when peribysin A was treated with tosyl chloride and triethylamine, an intramolecular S_N_2 reaction took place to form an ether ring. Dihydroxylation of the exo alkene, **105** yielded peribysin B bearing a vicinal diol. In contrast, when scandium(III) triflate, a Lewis acid, was employed in the presence of water, epoxide opening was accompanied to yield an allylic alcohol, peribysin C. Finally, when peribysin A was treated with an inorganic acid, the epoxide was opened to generate a more stable carbocation. Subsequent addition of water yielded peribysins F and G.

### 2.12. Synthesis of Thelepamide

In 2013, the Rodríguez group proposed the chemical structure of thelepamide for the first time after its isolation from the tidal zone-derived marine worm *Thelepus crispus* [108]. However, in 2018, it was revealed that there was an error in their stereochemical assignment of its structure, as indicated by total syntheses performed by the Rodríguez and Christmann groups [33]. The structure of thelepamide possesses unusual *N*,*O*-acetal and hemiacetal moieties, and it has been speculated that valine and cysteine may be involved in their biosyntheses due to the presence of a thioether and an isopropyl group.

**Scheme 14:**
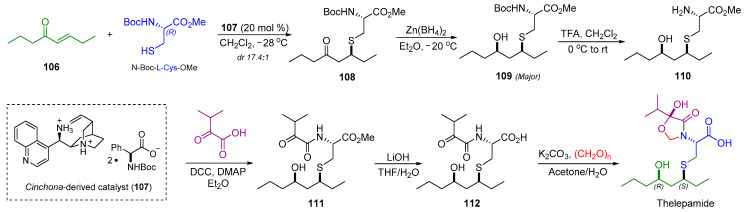
Synthetic route for preparation of the actual structure of thelepamide.

Their synthesis of thelepamide began with preparation of the chiral thioether through an enantioselective thia-Michael addition reaction with the aid of a *cinchona* alkaloid-derived catalyst, **107** [109]. Subsequently, a C-O bond was formed through a diastereoselective reduction of **108** to give the intended configuration of **109**. After recovery of the free amine of cysteine, this group was acylated with 2-ketoisovaleric acid. Finally, *N*,*O*-acetal formation was achieved by the addition of formaldehyde and sonication under basic conditions [110] (Scheme 14).

## 3. Discussion and Conclusions

The limitless resources provided by oceans are crucial to the discovery of new therapeutic ingredients. Molecules with unique and novel structures, obtained via novel combinations and arrangements of carbon, hydrogen, and heteroatoms, have been discovered in oceans, and substantial research efforts have been aimed at understanding these natural molecules. During this process, mistakes can sometimes prevent the accurate characterization of natural substances. Thus, errors in structure determination are frequently reported. Natural products originating from the sea are difficult to predict because they are classified broadly, and the structures are often complex. The structural misassignment is unfortunate and methods to avoid this can significantly reduce the workload and cost of the research.

Even with significant developments in tools for spectroscopic analysis over the past few years, mistakes are still made, and erroneous interpretations or premature convictions in the structural analysis of natural products have mostly been refuted through synthetic chemistry. The total synthesis of a molecular skeleton from a simple starting material is a time-consuming and tedious task. Therefore, the modification of material structures via total synthesis is essential. This way, an effective strategy for building the corresponding skeleton rather than simply correcting the structure can be developed. Thus, total synthesis will continue to be used as a reliable means for accurate structural identification and make significant contributions to helping humans unravel the mysteries of nature. The steady accumulation of efforts to discover and empirically manufacture substances created by nature and to reproduce artificial molecules with better pharmacological properties will ultimately help us overcome the current COVID-19 crisis that we are all suffering from.

## Figures and Tables

**Table 1:** Examples of marine natural products that have been corrected at a glance (from 2018 to 2021).

Entry	Name	Initially Proposed Structure	Revised Structure	Discrepancy	Ref.
	Molecular Class			Revision Clue	
1	Boholamide A	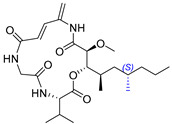	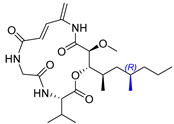	Incorrect stereochemistry	[15]
	Macrocyclic depsipeptide			Total synthesis	
2	Chrysamide B	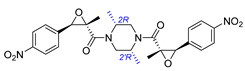	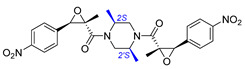	Incorrect stereochemistry	[16]
	Dimeric epoxyamide			Total synthesis	
3	Dysiherbol A	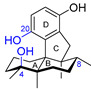	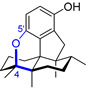	Incorrect formula	[17,18,19]
	Avarane meroterpene			Total synthesis	
4	Echinosulfone A	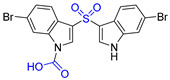	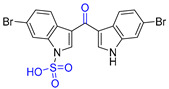	Incorrect formula	[20]
	*bis*-Indole alkaloid			Total synthesis	
5	Echinosulfonic acid A~D	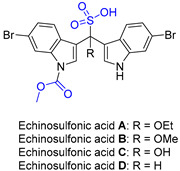	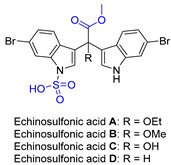	Incorrect formula	[21]
	*bis*-Indole alkaloid			Close inspection w/ plausible biosynthesis	
6	Haliclonadiamine	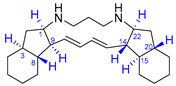	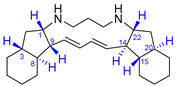	Incorrect stereochemistry	[22]
	*bis*-Indane			X-ray crystallography	
7	Halioxepine	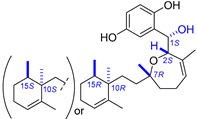	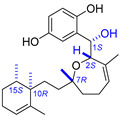	Incorrect stereochemistry	[23]
	Meroditerpene			Total synthesis	
8	Iriomoteolide-2a	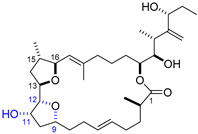	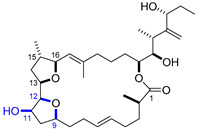	Incorrect stereochemistry	[24]
	23-Membered macrocycle			Total synthesis	
9	Julichromes Q_3.3_ and Q_3.5_	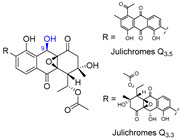	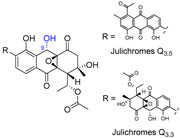	Incorrect stereochemistry	[25]
	Aromatic polyketide			X-ray crystallography	
10	Keramamide A and L	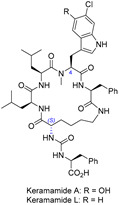	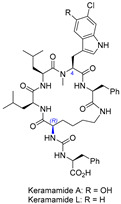	Incorrect stereochemistry	[26]
	Anabaenopeptin-type peptide			Total synthesis	
11	Mozamide A	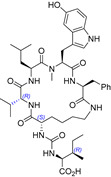	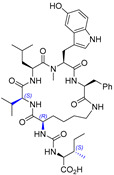	Incorrect stereochemistry	[27]
	Anabaenopeptin-type peptide			Total synthesis	
12	Mytilipin B	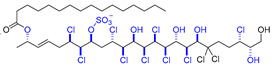	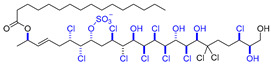	Incorrect stereochemistry	[28]
	Chlorosulfolipid			Total synthesis	
13	Nocarbenzoxazole G	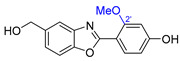	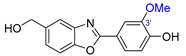	Incorrect constitution	[29]
	Nocarbobenzoxazole (2-arylbenzoxazole)			Total synthesis	
14	Solomonamide A and B	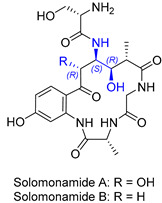	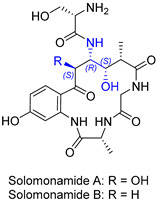	Incorrect stereochemistry	[30,31]
	Marocyclic peptide			Total synthesis	
15	Peribysins A, B, C, F, and G	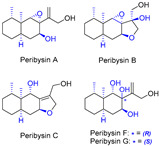	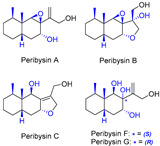	Incorrect stereochemistry	[32]
	Peribysin family			Total synthesis	
16	Thelepamide	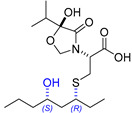	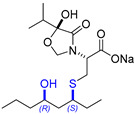	Incorrect stereochemistry	[33]
	Tetraketide -amino acid			Total synthesis	
17	Wentiquinone C	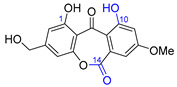	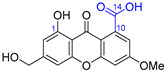	Incorrect constitution	[34]
	Xanthone			Chemical derivatization	

## Data Availability

Not applicable.
